# Transaortic valve replacement, complications and validation of a protocol in ICU

**DOI:** 10.1186/2197-425X-3-S1-A542

**Published:** 2015-10-01

**Authors:** A Berrazueta, C Carbajales, JM Ayuela-Azcarate, E Martinez Barrio, P Tejedor, E Portugal, M Valle, M Gero

**Affiliations:** Burgos University Hospital, Intensive Care, Burgos, Spain

## Introduction

Aortic stenosis(AS) is the most prevalent valvular disease (incidence 2% over 65years). Valvular area decreases 0.3m/s/y, transvalvular gradient (7mmHg/y) and blood speed(0.3m/s/y) increases with age. AS treatment can be medical or surgical:heart valve replacement surgery or percutaneous placement of an aortic endoprothesis(TAVR) in patients with high perioperative risk.TAVR is beneficial and improves their expectative and quality of life (QOL) compared with medical treatment.

## Objectives

To describe the incidence of complications of TAVR and to determine the validity of our protocol.

## Methods

Descriptive observational study during 2012-2014 of TAVR complications with Corevalve device at Burgos University Hospital.We analyzed epidemiological characteristics, comorbidities, and complications.We applied the actual protocol in all patients, which consist in femoral artery access, provisional pacemaker(PM), antibiotic and high digestive bleeding prophylaxis.During TAVR patients are superficially sedated and ventilated with self-expanded balloon. Afterwards patients are admitted to ICU continuing hemodynamic monitoring, neurologic vigilance, and vascular access care hourly. Systematically and detailed echocardiography is made.Double antiaggregation and haemoglobin are optimized. Seriated blood tests are taken. If there is no atrioventricular block(AVB) after 24 hours the PM is removed.

## Results

We analyze 22 patients with severe AS undergoing TAVR, with good QOL and comorbidities similar to those described in table 1. Mean age 84.2years(SD 5.063), 68.2% women. Median APACHE II score 13 and mean Euroscore 17.

**Table 1:** *Patient’s Comorbidities.*

Arterial Hypertension(AHT)	77%
Cardiac Insufficiency	54.5%
Atrial Fibrillation	45.5%
Coronary disease	45.5%
Acute Kidney Injury(AKI)	34.4%
Diabetes Mellitus	22.7%
Pulmonar Hypertension	22.7%
Obesity	9.1%
Artheriosclerosis	9.1%
Ictus	9.1%

Complications in ICU (Table 2): blood transfusion 63.7%, acute kidney injury(AKI) 31.8%, AVB 27.3%(definitive PM); stroke(9.1%), and absence of infection. Three patients had a cardiac arrest during the procedure(TAVR was not implanted) and only one of them survived.TAVR migration occurred once and required recolocation.

**Table 2:** *Complications after TAVR implantation.*

Residual Aortic Regurgitation	18.2%
Cardiac Arrest	18.2%
Valvular migration and prosthesis recolocation	4.2%
Malignant arrhythmias	4.2%
Pneumothorax	4.2%
Mechanical Ventilation	9.1%
ICU Stay	2.7 (SD 0.3)
Hospital Stay	11.23 (SD 1.36)
30 days Mortality	13.6%
1 year Mortality	13.6%

## Conclusions

TAVR using our protocol is a safe technique but not free of complications in patients with high surgical risk. Most of these complications were transfusion requirements, conduction system abnormalities, and AKI resolved at discharge. No nosocomial infections or need of prolonged mechanical ventilation was observed. These results are comparable to the experience in other centers, so we validate our protocol.
